# Nurse educators perceptions of simulation teaching in Chinese context: benefits and barriers

**DOI:** 10.7717/peerj.11519

**Published:** 2021-06-17

**Authors:** Dan Luo, Bing-Xiang Yang, Qian Liu, Aijing Xu, Yaxuan Fang, Ailing Wang, Sihong Yu, Ting Li

**Affiliations:** 1 School of Health Sciences, Wuhan University, Wuhan, China, China; 2 School of Nursing, Southern Medical University, Guangzhou, China

**Keywords:** Simulation, Nursing education, Faculty perception, Implementation

## Abstract

**Background:**

Although simulated teaching was introduced to China in the 1990s, it remains underused in nursing education. Determining how Chinese nurse educators feel about using simulation in their institutions is very important for faculty training and has the potential to influence simulation implementation.

**Method:**

This cross-sectional descriptive study was undertaken to identify the nurse educators’ experiences in the use of simulation from various regions of China. One hundred and thirty-six nurse educators provided demographic data and information about simulation implementation within their institutions and explored the perceived barriers and benefits of simulation usage.

**Results:**

The survey data shows that 108 participants have used simulation in their work, but less than 92 (67.6%) of the respondents had used this teaching strategy more than ten times in last year. The study identified four factors hindering nurse faculty from simulation adoption: (1) concerns with student readiness; (2) the need for faculty team-building for simulation teaching; (3) lack of adequate simulation resources; and (4) thoughtful integration of simulation into nursing curricula.

**Conclusions:**

Study data suggest that faculty training programs for simulation should be based on the nurse educators’ training needs, including systematically designed training topics, and the provision of hands-on learning simulation activities with expert feedback to help nurse educators achieve the competencies required for effective simulation-based education.

## Introduction

As an alternative educational pedagogy, simulation replicates a particular set of conditions to resemble authentic clinical (Khalaila, 2014). Simulation-based teaching has become more and more popular in health education. It provides an effective way for students to prepare for complicated clinical situations, especially for the low incidence high-risk events (Almeida et al., 2018), and it allows interdisciplinary teamwork, enables students and medical staff to cultivate communication skills, enhances their clinical practice in a nonthreatening setting (Norman, 2012). In nursing education, there are a plethora of studies support that both nurse faculty and students found simulation is valuable and beneficial to develop metacognitive thinking, improve students’ self-confidence and satisfaction of learning (Gaba, 2004; Jeffries, 2012; Yeun et al., 2014; Zhang et al., 2014; Lee et al., 2020). Furthermore, a longitudinal study conducted by the National Council of State Boards of Nursing (NCSBN) suggested that qualified simulated experience can efficiently replace up to 50% of clinical placements (Hayden et al., 2014). Simulation-based teaching becomes a vital teaching strategy in nursing education.

Simulation-based education was introduced into Chinese nursing education at the beginning of the 1990s (Wang, Fitzpatrick & Petrini, 2013b). Under the Chinese national nurse agenda for promoting a higher quality nursing education (Gao et al., 2017), many institutions and schools have integrated innovative simulation activities into their education programs and nursing curricula, moving from traditional teaching into student-centered education to better prepare graduates for the increasing complexities of health care environments (Xie, Jiang & Yin, 2015; Li, Wang & Liu, 2017). Moreover, the growing attention of the public to their health care rights restricts nursing students’ clinical practice opportunities (Park & Kim, 2000). As a result, an increasing number of nursing schools have considered applying more simulation in their curricula (Wang, Fitzpatrick & Petrini, 2013a; Wang, Sun & Wu, 2014a). Although nearly three decades have passed, many nurse educators continue to struggle to maximize the usage of simulation, facilitate students’ simulation preparation, conduct simulation and evaluate the outcomes of simulation experiences (Bogossian et al., 2018). Nursing faculty shortages, high workload demand, and insufficient funds delay the usage of simulation throughout nursing curricula. Moreover, there is a lack of qualified simulation facilitators in China (Li, Petrini & Stone, 2018).

The International Nursing Association for Clinical Simulation and Learning (INACSL) created standards of simulation facilitation with best practice, which indicates that a facilitator with well-developed simulation facilitation skills manages the complexity of simulation activities (Boese et al., 2013). In 2018, the National League for Nursing (NLN) implemented a China Faculty Development Program for Simulation to leverage the resources of Chinese nursing schools to lead Chinese nurse educators in the transformation of nursing education. This transformation would provide for the development of a safe and effective nursing workforce in China through leadership training and faculty development in the use of simulation in teaching and learning (Forneris, 2019). Considering that simulation education is the key to guiding students’ learning (Boese et al., 2013), determining how Chinese nurse educators feel about using simulation in their institutions is very important for faculty training and can influence simulation adoption. This study aimed to identify the nurse educators’ experiences of using simulation-based education from various regions of China, explore their perceptions and identify factors that influence the implementation of simulation in the current context of China.

*Research questions.* Research questions for this study were: (1) What is the current status of simulation teaching adoption by Chinese nursing educators? (2) What are the perceived barriers and benefits of simulation implementation from nursing instructors?

## Theoretical Framework

Built on the foundation of initial National League of Nursing (NLN)/Jeffries’ simulation framework (Jeffries, 2005), the NLN-Jeffries Simulation Theory is a theoretical framework comprising seven conceptual components: context, background, design, simulation experience, facilitator and educational strategies, participant and outcomes (Hallmark, Thomas & Gantt, 2014). The NLN recommends using the theory framework to guide simulation activities (Adamson, 2015). Facilitator characteristics, such as the educators’ experiences, teaching ability, nursing competence, and technological skills, are aspects that influence the educator’s ability to facilitate and evaluate simulation (Boese et al., 2013; Jeffries, 2016). In this study, the National League for Nursing-Jeffries Simulation Theory provided the framework for gathering faculty’s opinions in this survey.

## Materials & Methods

### Study design

A cross-sectional descriptive study was used to explore Chinese nurse educators’ perceptions of simulation usage in the undergraduate nursing program. In this study, a quantitative study design was used to guide the whole process, and qualitative data analysis method was used when dealing with the open-end questions.

### Sampling and setting

A convenience sample of 136 nurse educators attending a simulation-based teaching workshop at a large university in Wuhan, China, were asked to complete a questionnaire at the beginning of the workshop. These educators came from 28 different provinces in China. Orally consent was gained from participants. They were informed that their participation was voluntary, and they could refuse to complete the questionnaire without prejudice. No identifying information was obtained. The Medical department of Wuhan University approval to carry out the study within its facilities (2021YF0002).

### Data collection tool

A structured questionnaire was developed based on National League for Nursing-Jeffries framework (Hallmark, Thomas & Gantt, 2014) and literature review. The questionnaire was used to obtain demographic data and information about simulation implementation within participants’ institution, and to explore the perceived barriers and benefits of simulation usage. The questionnaire consisted of nine closed-ended items and two open-ended questions. The closed-ended items asked for information about the nurse educators’ demographics, teaching background, educational practice background, level of nursing students being taught, and their use of simulation in teaching. Two open-ended questions explored the faculty’s perceived benefits and barriers to implementing simulation.

Three nursing educators at the researcher’s institution were asked to review the questionnaire for face validity to ensure that the questionnaire covered the content and its purpose. All reviewers were recognized as experts in simulation-based education. They agreed that the questionnaire appeared sound and relevant, and the purpose was deemed logically consistent with the research objectives.

### Statistical analysis

Descriptive statistics, including percentage and mean with standard deviation, were used to describe participants’ demographic data and their adoption of simulation in the nursing institution where they were employed. Independent *t*-test and Spearman correlation were used to explore the impact of factors related to the implementation of simulation. Regression analysis was used to identify the predictors of the application of simulation teaching based on the correlation results. Inductive thematic analysis was used to identify perceptions of the nurse educators’ self-reported barriers and benefits in the open-ended questions.

## Results

In total, 136 participants completed the questionnaire, and 131 (96.3%) were female. Sixty-nine (50.7%) had a master’s degree, sixty-one (44.9%) held a bachelor’s degree, three (2.2%) had a diploma, and three (2.2%) were doctoral degrees /doctoral candidates. The age of participants ranged from 25 to 58 years old (36.67 ± 7.76), and the average work experiences ranged from 0 to 33 years (9.75 ± 7.90).

### Implementation of simulation in the nursing program

**Figure 1:**
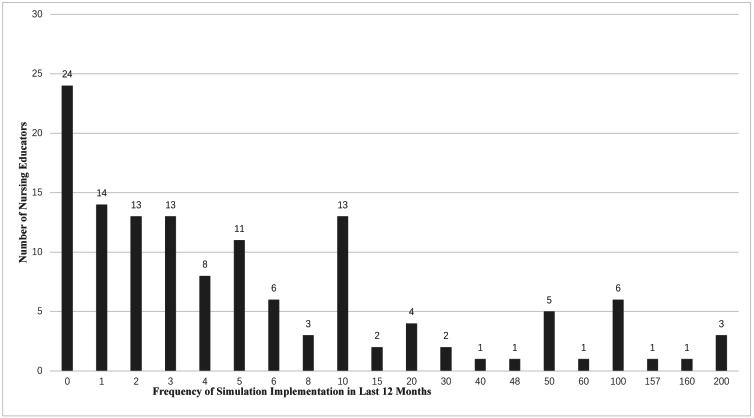
Self-reported frequency of simulation usage in the previous12 months.

The self-reported implementation of simulation shows that 108 (79.4%) of participants have used simulation in their work, while other participants reported they have never used it. The frequency of use of simulation in the previous 12 months ranged from 0 to 200 times. Figure 1 details the frequency of the use of simulation.

Seventy-five participants responded to the question that focused on what hinders them from adopting simulation in their working setting: 34 (45.33%) stated that they did not know how to run a scenario; 24 (15.4%) think that their courses were not appropriate for simulation, seven (9.33%) considered that simulation was too complex to implement, and the others (13.3%) reported reasons such as “*students do not adapt [sic]*” and “*I do not like design scenario [sic]*.” Compared with the reasons why they plan to use simulation in the future, 131 participants responded as follows: 63 (48.09%) stated that there is a need for simulation in their course, 39 (29.77%) showed interest in using simulation-based teaching, 17 (12.98%) reported that they followed their institution’s requirements, and the remaining (9.2%) reported implementing simulation in teaching research and course reform activities.

### Impact of factors related to the implementation of simulation-based teaching

Independent *t*-test was performed to identify if there were significant differences between different groups of participants in regards to their frequency of simulation adoption in the previous 12 months. The result showed that there was no statistical difference between the different training experiences (*t* =  − 0.011, *p* = 0.991), participants educational background (*t* = 1,746, *p* = 0.084) and level of students being taught (*t* = 1.408, *p* = 0.162) in the amount of simulation adoption in previous 12 months.

The Spearman correlation analysis showed that the participants’ years of teaching (*r* = 0.24, *p* = 0.006) and the duration of simulation acquaintance (*r* = 0.444, *p* = 0.000) had a positive relationship with the frequency of simulation adoption in the previous12 months.

Stepwise linear regression was used to explore the predictors among the variables which correlated with the frequency of simulation adoption in the previous 12 months. An educator’s duration of simulation acquaintance is the only variable that affects the equation (*B* = 3.766, SE = 0.914, =0.34, *t* = 4.12, *P* < 0.000), and the R square of the equation was 0.116. The result shows that an educator’s duration of simulation acquaintance was a predictor of the frequency of simulation adoption.

#### Inductive thematic analysis of statements in open-ended questions

In this study, 107 participants answered questions about the barriers and benefits of simulation implementation. Their responses were coded using NVIVO 11.0 for inductive thematic analysis. In reflecting on the barriers to adopting simulation, the main themes, including barriers from students and faculty, resources, and curriculum integration. Themes identified as benefits containing enhancing student learning outcomes and motivating the learning process.

#### Perceived benefits

Twenty-two participants described the benefits of implementing simulation. The main themes included: good for students’ learning outcomes and motivation for students’ learning. The description of benefits for students’ learning outcomes was described as *“students could learn clinical skills better in simulation than traditional teaching”, “students could actively learn the knowledge”,* and *“students are well engaged in simulation teaching and learn well”.*

Other statements pointed out that simulation-based teaching could motivate students in the learning process. This theme was described as *“students love simulation”, “simulation could stimulate students learning, make them participate well”, “previously students do not have the opportunity to practice without fear of harming patients as simulation, so they like this teaching method”,* and *“students could have fun in simulation-based teaching”.*

### Perceived barriers

#### Concerns with student readiness

There were 47 (17.99%) participant statements that reported barriers concerning students’ readiness for simulation learning. Statements included *“students do not prepare well before simulation they do not want to cooperate in teaching activities”. ”The difference in students’ competency makes them not discuss well in the post-conference”. “Simulation-based teaching requires high competency, which our students could not meet”. “Some students could not respond in the scenario well”.* Nurse educators noticed that poorly prepared students showed negative attitudes during the simulation procedure. The related statements were: *“students do not think they prefer simulation”.* And *“they can’t engage in the simulation learning”.*

#### The need for faculty team-building for simulation teaching

Thirty-seven (12.6%) participants provided feedback stating that the barriers from simulation team-building influenced their implementation of simulation. More than eighty percent of them (83%) stated that *“simulation-based teaching requires a higher capacity of faculty …”* but their *“faculty members lack experiences of simulation-based teaching”.* There are *“too many students in our school…“* and *“It is hard to have small class size while teaching”.* They *“need more faculty”* and *“…faculty need more experience and knowledge to handle simulation-based teaching”.* They emphasized that the *”structure of the faculty team is not sufficient for simulation teaching…”* They have *“few opportunities for training…” “Few of them only received some training from the company…”* and they feel *“…faculty team lack of training experience in simulation operation”.*

#### Lack of adequate simulation resources

Twenty-six participants’ (9.54%) elaborated on the barriers caused by a lack of experimental resources. Most of the statements described these barriers as *“the teaching building is not large enough”,* and *“. . . basic resources are not enough, for example, the one-side visible glass”. “We don’t have high fidelity manikins”.* They are *“…lack of designed simulation scenarios”* and *“…lack of financial support for simulation teaching”.*

#### Thoughtful integration of simulation into nursing curricula

Seven of the participants mentioned barriers related to curriculum integration. These barriers were described as *“simulation teaching is time-consuming”, “my teaching content could not use simulation teaching”. “Sim-man could not perform as a real patient”,* and they feel *“…hard to perform communication in a simulation scenario”.*

## Discussion

Nursing educators are constantly striving to advance nursing education through innovative technology to prepare future nurses for the complexities of the health- care environments (Jansen et al., 2009). Through the integration of simulation into nursing education programs, the goal of providing quality education that enhances students’ learning outcomes could be partially achieved (Hayden, 2010; Warren et al., 2016). This study reports the survey findings relating to Chinese nurse educators’ perceptions in the adoption of simulation, with a focus on the factors that influence the use of simulation.

Participants of the survey mentioned that faculty show growing interests in the adoption of simulation in teaching, and an increasing number of nursing schools have purchased or are planning to use this innovative approach (Wang, Sun & Wu, 2014a; Zhang, 2017). The majority of respondents positively indicate that simulation served to increase students learning satisfaction, better motivate students’ active learning, and promote learning outcomes. Simulation spans the gap between theories and practice by creating a safe clinical learning context (O’Donnell et al., 2014). This literature supports the consensus of the advantages of simulation pedagogy. Simulation pedagogy contributes to the acquisition of students’ knowledge and skills and strengthens their metacognitive abilities (Hayden et al., 2014; Lam’e & Dixon-Woods, 2020; O’Donnell et al., 2014).

Although faculty show a generally supportive attitude towards simulation, their frequency of using simulation is not high (Nehring & Lashley, 2004). Simulation has not been fully used by nursing educators in China (Anderson et al., 2012). Our study results show that less than 92(67.6%) of the respondents had used this teaching method frequently, which means use simulation in their teaching less than ten times in the last year. Compared with existing studies that reported inadequate financial support, time and technology as the major obstacles to the usage of simulation in nursing education (Al-Ghareeb & Cooper, 2016; Cuchna, Walker & Van Lunen, 2019), the survey data reflects that more faculty struggle in helping students adapt to simulation learning, enhancing student engagement, and integrating simulation into the nursing course and curriculum. In the process of using simulation, they experienced a lack of team support. Instructor development was a chief obstacle to the full adoption of simulation in nursing education (Taibi & Kardong-Edgren, 2014). Educator preparedness in simulation teaching can influence the learners’ readiness for learning and the learning outcomes (Cockerham, 2015). Educator development in simulation should include a solid understanding of the principles of constructivism and learner-centered instruction as a foundation for simulation pedagogy (Taibi & Kardong-Edgren, 2014; Boese et al., 2013). Periodic faculty development programs for continuous simulation quality improvement are also needed to acknowledge ongoing faculty skills (Taibi & Kardong-Edgren, 2014).

Our study indicates that the respondents’ prior simulation training experiences haven’t changed their real application of simulation. Survey findings support the research of Arthur, Levett-Jones & Kable (2013) and King et al. (2008), which indicated that faculty had positive intentions to use simulation, but negative beliefs regarding the real application. An additional study pointed out that there were no significant differences in using the simulation technology pre and post attending the training program (Jones, Fahrenwald & Ficek, 2013). One of the possible reasons is that lots of Chinese educators have not been trained adequately. Several participants reflect that they have attended some seminars or workshops. Most of these training are one-time offerings, and some are sporadic. The information provided can help them gain a quick understanding of simulation teaching, but this knowledge has a different focus, not systematic enough to support their actual implementation (Cheng et al., 2015). A few educators reported that they have only received the training from manikin companies.

In the survey, some educators reported that they dare not try simulation because they had no hands-on learning activities in simulation training. These findings are consistent with the literature (Katz, Peifer & Armstrong, 2010; Jones, Fahrenwald & Ficek, 2013). In China, most of the simulation training programs focused primarily on the technical aspects of the simulation operation with little regard for the qualification of the simulation instructors (Cheng et al., 2015; Issenberg, 2006). Appropriate content selection to meet the pressing needs of faculty development can make the simulation instructor training more effective and promote the use of simulation (Topping et al., 2015). Taibi & Kardong-Edgren (2014) conducted an online survey to explore the training needs of simulation. They reported that the training priorities for simulation instructors include facilitating interprofessional communication, followed by guiding a debriefing session and integrating the simulation pedagogy into nursing curricula. They also indicated that the lowest need was mechanically operating a simulator. In their study, the faculty expressed that they would try simulation teaching with more support.

Recently, many Chinese faculty members realized that the use of simulation in nursing education requires additional knowledge and preparation (Shen et al., 2019). They have started to improve their simulation competencies in various ways (Forneris, 2019). But our survey results indicated that when using the simulation with students, a lot of faculty members had a lack of confidence regarding their own level of proficiency with simulation facilitation. By comparison, educators who used simulation teaching more frequently in the past 12 months showed stronger self-confidence. The faculty would gain confidence with the increased use of simulation (Katz, Peifer & Armstrong, 2010). The literature proposes that the persistent deliberate practice is an efficient way to develop necessary simulation teaching skills (Topping et al., 2015). Some researchers suggested that preferred strategies to develop simulation competencies were: (1) observing skilled instructors’ simulation teaching and understanding simulation through trial and error (Bentley & Seaback, 2011; Anderson et al., 2012); or (2) practicing simulation teaching in a safe and simulated educational environment with feedback from experienced simulation facilitators (Waxman & Telles, 2009). Some researchers emphasize that an overall training program of simulation-based education may promote competences of nursing educators (Roh, Kim & Issenberg, 2019; Topping et al., 2015).

## Conclusions

Although simulated teaching was introduced to China in the late 90s, it remains underused in nursing education (Zhu & Zhu, 2019). Many Chinese nursing schools have begun to realize the necessity of simulation-based education and are now scrambling to build simulation center/laboratories. In contrast, the training of simulation instructors is still in its early stages (Wang, Sun & Wu, 2014b). The quality of nursing simulation varies due to the lack of qualified instructors, and insufficiency of simulation teaching standards and the qualification of certification for simulation instructors exacerbates the situation. Given that many nursing faculty hope to master simulation teaching ability and improve their teaching quality to realize the students learning outcomes by participating in simulation training (Taibi & Kardong-Edgren, 2014), our study suggests that formal faculty training programs should be based on the educators’ training needs, systematically design the training topics, and provide hands-on learning simulation activities with expert feedback to help nursing educators enhance competencies required for simulation teaching progressively.

### Limitation

This study has a relatively limited number of respondents, and most of the targeted population are Chinese simulation users, preventing the generalization of the results. However, setting the Chinese simulated teaching users as the target group helps to understand in the context of Chinese nursing education and how to help faculty effectively increase the use of simulated teaching. Another limitation is the narrow focus of the descriptive study. Further study needs to be applied to address the details of Chinese educators’ training needs in simulation and how to gradually promote educators’ competencies with the growing level of educators.

##  Supplemental Information

10.7717/peerj.11519/supp-1 Supplemental Information 1 Raw data

10.7717/peerj.11519/supp-2 Supplemental Information 2 Questionnaire in Chinese

10.7717/peerj.11519/supp-3 Supplemental Information 3 Questionnaires used in this study

## Additional Information and Declarations

### Data Availability

The following information was supplied regarding data availability: Raw data are available as a Supplemental File.

## References

[ref-1] Adamson K (2015). A systematic review of the literature related to the NLN/Jeffries simulation framework. Nursing Education Perspectives.

[ref-2] Al-Ghareeb AZ, Cooper SJ (2016). Barriers and enablers to the use of high-fidelity patient simulation manikins in nurse education: an integrative review. Nurse Education Today.

[ref-3] Almeida R, Jorge BM, Souza-Junior VD, Mazzo A, Martins J, Negri EC, Mendes I (2018). Trends in research on simulation in the teaching of nursing: an integrative review. Nursing Education Perspectives.

[ref-4] Anderson M, Bond ML, Holmes TL, Cason CL (2012). Acquisition of simulation skills: survey of users. Clinical Simulation in Nursing.

[ref-5] Arthur C, Levett-Jones T, Kable A (2013). Quality indicators for the design and implementation of simulation experiences: a Delphi study. Nurse Education Today.

[ref-6] Bentley R, Seaback C (2011). A faculty development collaborative in interprofessional simulation. Journal of Professional Nursing.

[ref-7] Boese T, Cato M, Gonzalez L, Jones A, Kennedy K, Reese C, Decker S, Franklin AE, Gloe D, Lioce L, Meakim C, Sando CR, Borum JC (2013). Standards of best practice: simulation standard V: facilitator. Clinical Simulation in Nursing.

[ref-8] Bogossian F, Cooper S, Kelly M, Levett-Jones T, McKenna L, Slark J, Seaton P (2018). Best practice in clinical simulation education- are we there yet? A cross-sectional survey of simulation in Australian and New Zealand pre-registration nursing education. Collegian.

[ref-9] Cheng Y, Liu H, Zhang A, Liang Y, Yang Q, Wu X (2015). Qualitative research about evaluation on scenario simulated teaching training among clinical nursing faculty. Medicine Teaching in University.

[ref-10] Cockerham ME (2015). Effect of faculty training on improving the consistency of student assessment and debriefing in clinical simulation. Clinical Simulation in Nursing.

[ref-11] Cuchna JW, Walker SE, Van Lunen BL (2019). Simulations and standardized patients in athletic training: part 2 athletic training educators’ perceived barriers to use. Athletic Training Education Journal.

[ref-12] Forneris SG (2019). One belt one road: bringing the NLN together with nursing education leaders from China. Nursing Education Perspectives.

[ref-13] Gaba DM (2004). The future vision of simulation in health care. Quality and Safety in Health Care.

[ref-14] Gao Y, Zhang P-P, Wen S-F, Chen Y-G (2017). Challenge, opportunity and development: influencing factors and tendencies of curriculum innovation on undergraduate nursing education in the mainland of China. Chinese Nursing Research.

[ref-15] Hallmark BF, Thomas CM, Gantt L (2014). The educational practices construct of the NLN/Jeffries simulation framework: state of the science. Clinical Simulation in Nursing.

[ref-16] Hayden J (2010). Use of simulation in nursing education: national survey results. Journal of Nursing Regulation.

[ref-17] Hayden JK, Smiley RA, Alexander M, Kardong-Edgren S, Jeffries PR (2014). The NCSBN national simulation study: a longitudinal, randomized, controlled study replacing clinical hours with simulation in prelicensure nursing education. Journal of Nursing Regulation.

[ref-18] Issenberg SB (2006). The scope of simulation-based healthcare education. Simulation in Healthcare : Journal of the Society for Simulation in Healthcare.

[ref-19] Jansen DA, Johnson N, Larson G, Berry C, Brenner GH (2009). Nursing faculty perceptions of obstacles to utilizing manikin-based simulations and proposed solutions. Clinical Simulation in Nursing.

[ref-20] Jeffries PR (2005). Designing simulations for nursing education. Annual review of nursing education.

[ref-21] Jeffries PR (2012). Simulation in nursing education: from conceptualization to evaluation.

[ref-22] Jeffries PR (2016). The NLN Jeffries simulation theory.

[ref-23] Jones AL, Fahrenwald N, Ficek A (2013). Testing Ajzens theory of planned behavior for faculty simulation development. Clinical Simulation in Nursing.

[ref-24] Katz GB, Peifer KL, Armstrong G (2010). Assessment of patient simulation use in selected baccalaureate nursing programs in the united states. Simulation in Healthcare.

[ref-25] Khalaila R (2014). Simulation in nursing education: an evaluation of students’ outcomes at their first clinical practice combined with simulations. Nurse Education Today.

[ref-26] King CJ, Moseley S, Hindenlang B, Kuritz P (2008). Limited use of the human patient simulator by nurse faculty: an intervention program designed to increase use. International Journal of Nursing Education Scholarship.

[ref-27] Lam’e G, Dixon-Woods M (2020). Using clinical simulation to study how to improve quality and safety in healthcare. BMJ Simulation & Technology Enhanced Learning.

[ref-28] Lee J, Lee H, Kim S, Choi M, Ko IS, Bae J, Kim SH (2020). Debriefing methods and learning outcomes in simulation nursing education: a systematic review and meta-analysis. Nurse Education Today.

[ref-29] Li T, Petrini MA, Stone TE (2018). Baccalaureate nursing students’ perspectives of peer tutoring in simulation laboratory, a q methodology study. Nurse Education Today.

[ref-30] Li T, Wang A, Liu Y (2017). The use of concept map in high fidelity simulation to teach baccalaureate nursing specialty course. Journal of Nursing Science.

[ref-31] Nehring WM, Lashley FR (2004). Current use and opinions regarding human patient simulators in nursing education: an international survey. Nursing Education Perspectives.

[ref-32] Norman J (2012). Systematic review of the literature on simulation in nursing education. ABNF Journal.

[ref-33] O’Donnell JM, Decker S, Howard V, Levett-Jones T, Miller CW (2014). NLN/Jeffries simulation framework state of the science project: simulation learning outcomes. Clinical Simulation in Nursing.

[ref-34] Park M-Y, Kim S-Y (2000). A qualitative study of nursing students’ first clinical experience. The Journal of Korean Academic Society of Nursing Education.

[ref-35] Roh YS, Kim M, Issenberg SB (2019). Perceived competence and training priorities of Korean nursing simulation instructors. Clinical Simulation in Nursing.

[ref-36] Shen L, Yang J, Jin X, Hou L, Shang S, Zhang Y (2019). Based on Delphi method and analytic hierarchy process to construct the evaluation index system of nursing simulation teaching quality. Nurse Education Today.

[ref-37] Taibi DM, Kardong-Edgren S (2014). Health care educator training in simulation: A survey and web site development. Clinical Simulation in Nursing.

[ref-38] Topping A, Bøje RB, Rekola L, Hartvigsen T, Prescott S, Bland A, Hope A, Haho P, Hannula L (2015). Towards identifying nurse educator competencies required for simulation-based learning: a systemised rapid review and synthesis. Nurse Education Today.

[ref-39] Wang AL, Fitzpatrick JJ, Petrini MA (2013a). Comparison of two simulation methods on Chinese BSN students learning. Clinical Simulation in Nursing.

[ref-40] Wang AL, Fitzpatrick JJ, Petrini MA (2013b). Use of simulation among Chinese nursing students. Clinical Simulation in Nursing.

[ref-41] Wang Y, Sun L, Wu Y (2014a). Current situation and reflection of simulation teaching in nursing education in china. Chinese Nursing Management.

[ref-42] Wang Y, Sun L, Wu Y (2014b). Current situation and reflection of simulation teaching in nursing education in china. Chinese Nursing Management.

[ref-43] Warren JN, Luctkar-Flude M, Godfrey C, Lukewich J (2016). A systematic review of the effectiveness of simulation-based education on satisfaction and learning outcomes in nurse practitioner programs. Nurse Education Today.

[ref-44] Waxman K, Telles CL (2009). The use of Benner’s framework in high-fidelity simulation faculty development: the bay area simulation collaborative model. Clinical Simulation in Nursing.

[ref-45] Xie X, Jiang L, Yin Z (2015). Application of comprehensive high fidelity simulation in teaching of hospital emergency and critical care. Chinese Journal of Nursing Science.

[ref-46] Yeun EJ, Bang HY, Ryoo EN, Ha E-H (2014). Attitudes toward simulation-based learning in nursing students: an application of q methodology. Nurse Education Today.

[ref-47] Zhang J (2017). Perceptions of simulation-assisted teaching among baccalaureate nursing students in Chinese context: benefits, process and barriers. Journal of Professional Nursing.

[ref-48] Zhang C, Miller C, Volkman K, Meza J, Jones K (2014). Evaluation of the team performance observation tool with targeted behavioral markers in simulation-based interprofessional education. Journal of Interprofessional Care.

[ref-49] Zhu X, Zhu X (2019). Investigation and analysis on medical simulation.

