# Supplementary material for: Nurse educators perceptions of simulation teaching in Chinese context: benefits and barriers
Source: PeerJ. 2021 Jun 17;9:e11519. doi: 10.7717/peerj.11519 (PMC8214848; doi:10.7717/peerj.11519)
Supplement: Supplemental Information 2 [file peerj-09-11519-s002.doc]

**关于模拟教学教师观点的调查**

请回答下列问题或在下列问题的答案中进行选择，在答案后面的方框中打√。如果您不确定怎么回答，请给出您认为的最合适的答案。谢谢！

1. 年龄： 岁
2. 性别：□1. 男 □2. 女
3. 您的学历是：

□1.大专 □2.本科 □3.硕士 □4.博士 □5.在职博士 □6.其他请注明

1. 您所教的学生的层次：（选择所有适用的）

1. □本科生 2. □硕士研究生 3. □博士研究生

4. □成教学生 5. □其他 （请注明：_________________________）

1. 您从事教学工作 年
2. 您接触模拟教学 年
3. 您是否接受过模拟教学的相关培训？

1. 是 □ 0. 否 □

如果否，请问您不喜欢的原因是 。

1. 您是否曾在您所教授的课程中使用模拟教学？

1. 是 □ 0. 否 □

1. 在过去的一年中，请问您使用模拟教学的次数大概是 次。
2. 请分享您在使用模拟教学中的感受到的能促进模拟的因素：

.

.

1. 对于模拟教学的使用，您觉得您面临的最大挑战是什么？

.
