# Supplementary material for: Nurse educators perceptions of simulation teaching in Chinese context: benefits and barriers
Source: PeerJ. 2021 Jun 17;9:e11519. doi: 10.7717/peerj.11519 (PMC8214848; doi:10.7717/peerj.11519)
Supplement: Supplemental Information 3 [file peerj-09-11519-s003.doc]

Survey of teacher's opinion of simulation teaching

Please answer the following questions or choose the answers to the following questions, and tick the box after the answer. If you are not sure how to answer, please choose the most appropriate answer. Thank you!

1. Age： years
2. Sex：□1. F □2. M
3. Education level：

□1.Diploma □2.Bacholar □3.Master □4.Doctor candidate □5.Doctorate □6.other

1. Your students’ level

□ Undergraduate □Master degree □Doctorate students □ Adult Education Students □Others (please specify: )

1. Years of your teaching : years
2. Years since you know simulation: years
3. Have you received any training related to simulation teaching?

1. Yes □ 0. No □

1. Have you ever used simulation teaching in the courses you teach?

1. Yes □ 0. No □

1. In the past year, how many times of simulation-based teaching have you used? Times
2. Please share your perceived facilitators in using simulated teaching

11. For the use of simulation teaching, what are the biggest challenges you face?
